# Supplementary material for: Reconfigurable optomechanical circulator and directional amplifier
Source: Nat Commun. 2018 May 4;9:1797. doi: 10.1038/s41467-018-04187-8 (PMC5935678; doi:10.1038/s41467-018-04187-8)
Supplement: Supplementary file 1 — Supplementary Information [file 41467_2018_4187_MOESM1_ESM.pdf]

Supplementary Information:
Reconfigurable optomechanical circulator and directional amplifier

Shen et al.

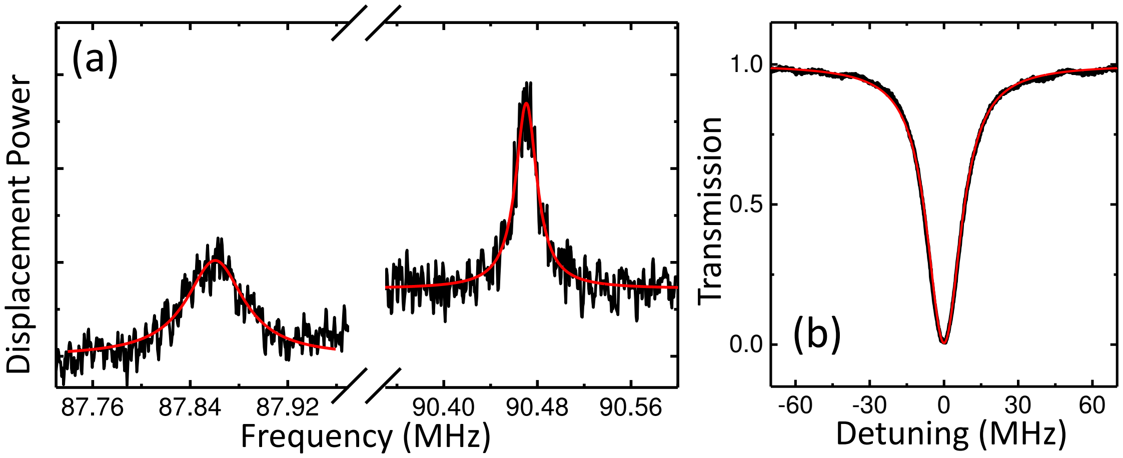

Supplementary Figure 1: **(a) Displacement power spectra of the two radial breathing modes obtained from a spectrum analyser. (b) Optical transmission spectrum for the optical mode. The red line is the result of calculation using the parameters $\kappa_\alpha/2\pi = 9\,\mathrm{MHz}$, $\kappa_\beta/2\pi = 4.2\,\mathrm{MHz}$, $\kappa_0/2\pi = 3\,\mathrm{MHz}$, and $J/2\pi = 1.5\,\mathrm{MHz}$.**

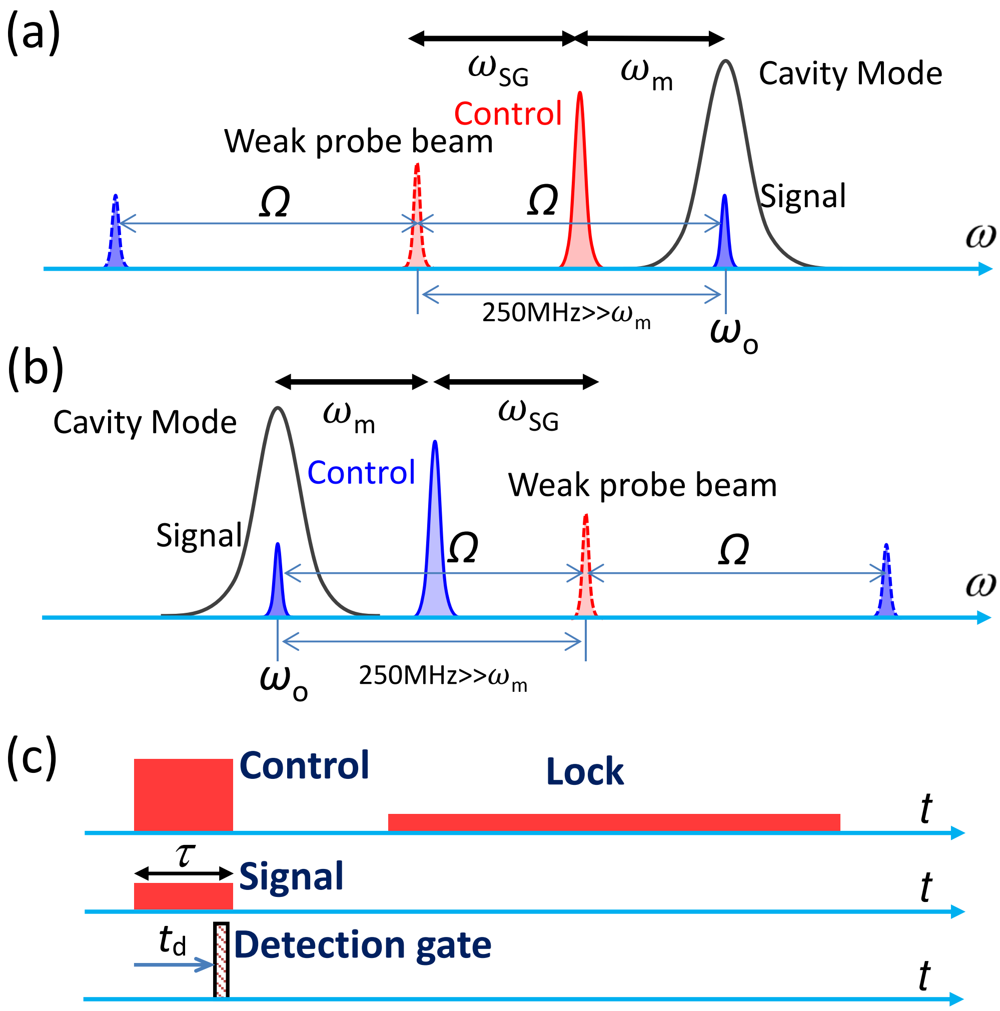

Supplementary Figure 2: Spectral positions and pulse sequences. (a) To demonstrate the circulator, the control field is locked at a frequency that is red-detuned from the optical mode by one mechanical frequency ω_m. The weak probe beam is also red detuned from the optical mode by 250 MHz. The signal pulse is generated by sending the weak probe beam through an EOM with modulation frequency Ω. (b) To demonstrate the directional amplifier, the control field is locked at a frequency that is blue-detuned from the optical mode by one mechanical frequency. (c) Schematic of the pulse sequences and detection gate used for the transient experiments.

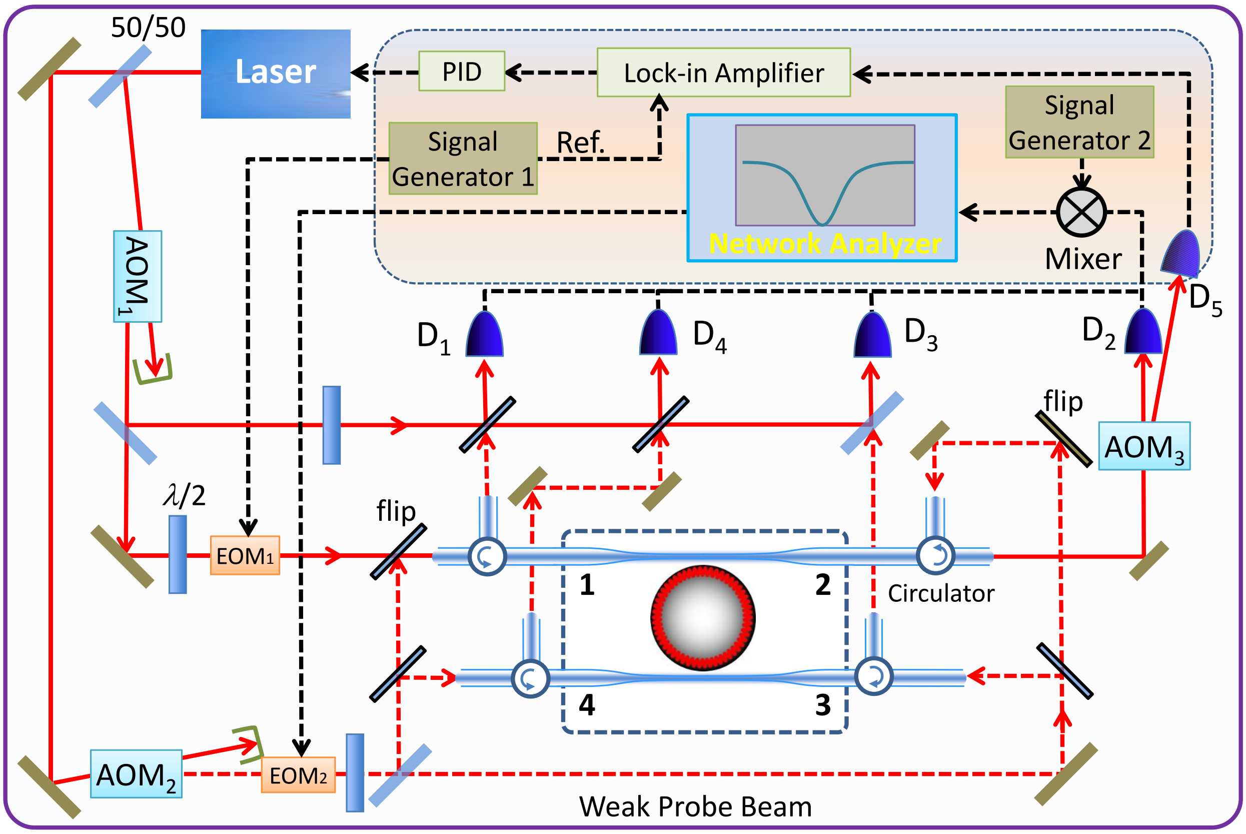

Supplementary Figure 3: Detailed experimental setup. Red lines represent optical paths, and black lines represent electrical connections. AOM, EOM, PID, D, and $\lambda/2$ represent the acoustic-optic modulator, electro-optic modulator, proportional–integral–derivative controller, photo-detector, and half-wave plate, respectively.

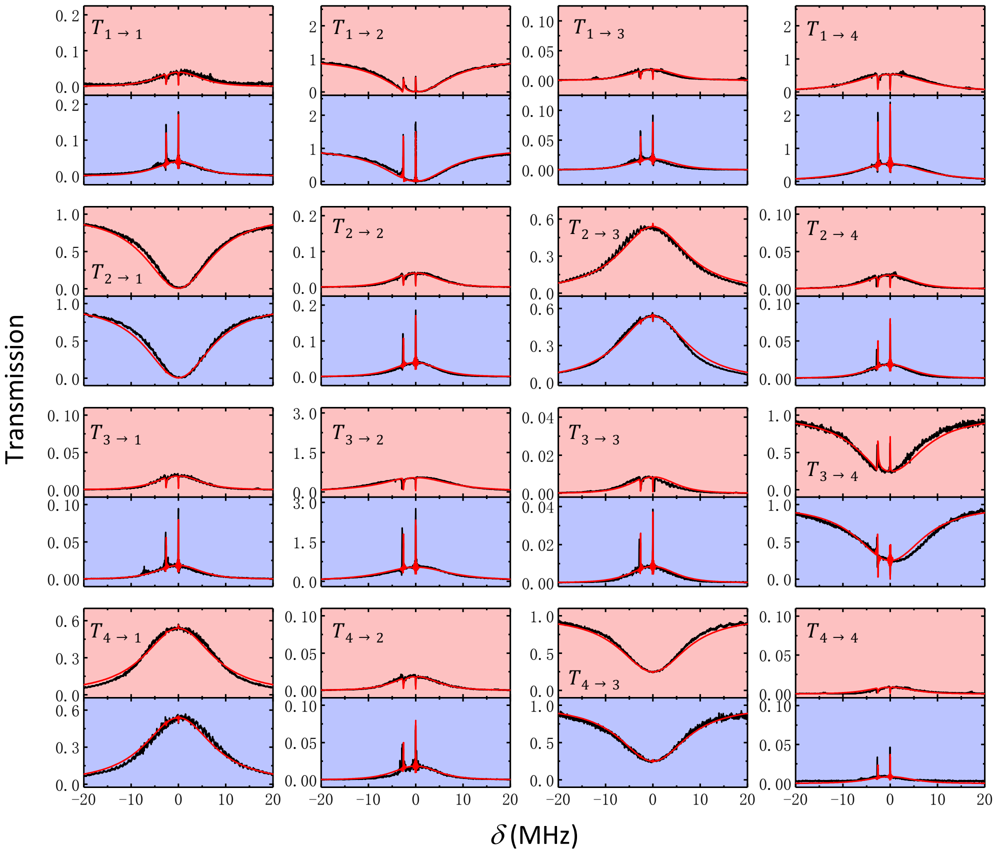

Supplementary Figure 4: Transmission spectra. For each port-to-port transmission spectrum $T_{i\to j}$, the upper panel shows experimental results for a red-detuned control field and the lower panel corresponds to a blue-detuned control field. The red lines are the results of calculations using the parameters $\kappa_\alpha/2\pi = 9$ MHz, $\kappa_\beta/2\pi = 4.2$ MHz, $\kappa_0/2\pi = 3$ MHz, $J/2\pi = 1.5$ MHz, $C_{\mathbf{cw}} = -1.7$ and 3.4, and $C_{\mathbf{cw}}/C'_{\mathbf{cw}} = 2.28$.

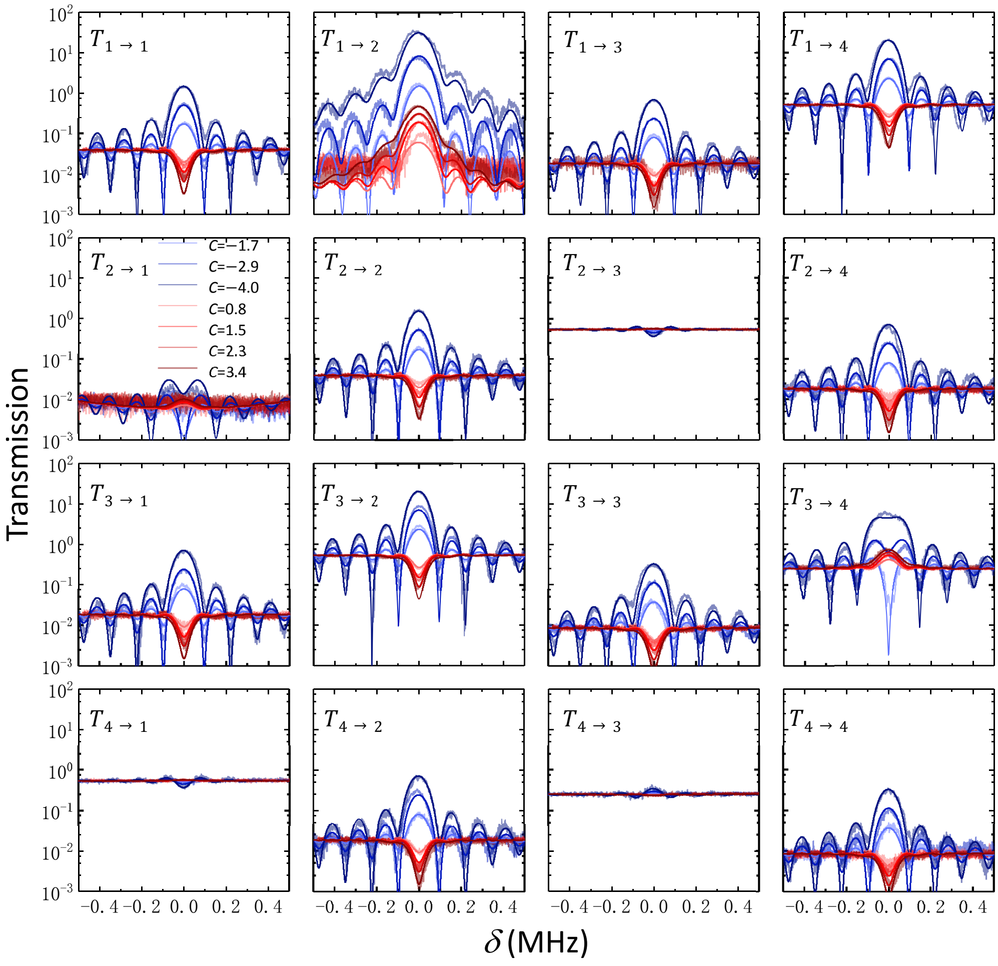

Supplementary Figure 5: **Detailed transmission spectra. Detailed transmission spectra around $\delta \sim 0$ for different control powers with a red-detuned control field (red lines) and a blue-detuned control field (blue lines). The darker lines are the results of calculations using $C_{\mathbf{cw}} = -1.7,\ -2.9,\ -4,\ 0.8,\ 1.5,\ 2.3,$ and 3.4.**

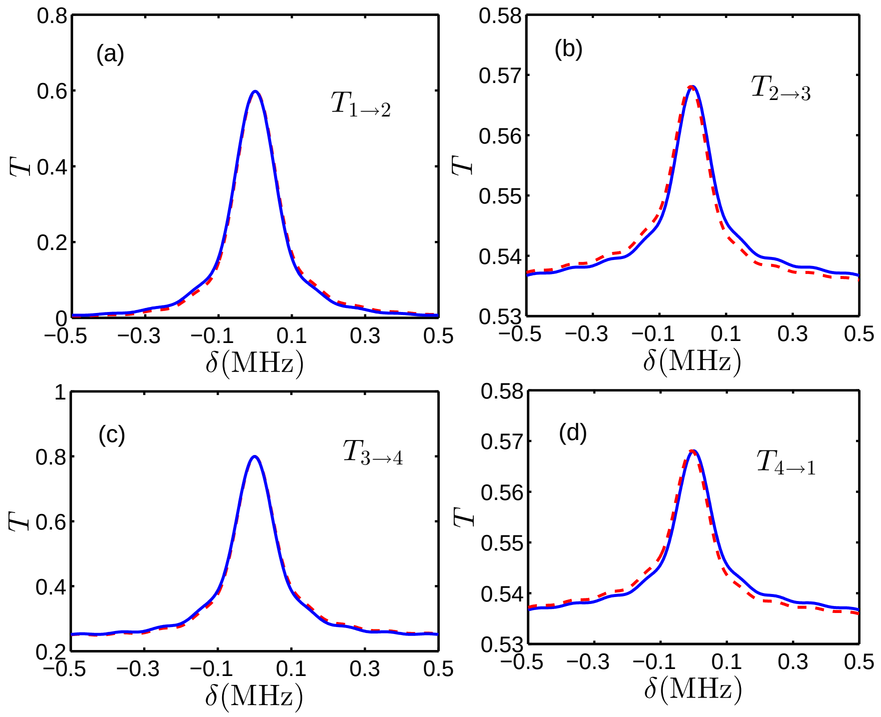

Supplementary Figure 6: **(a-d) Transmittance $T_{i\to i+1}$ as a function of the signal detuning $\delta = \omega - \omega_{\rm o}$. The red dashed-lines show the transmittance matrix including the back-scattering and two mechanical modes. The blue solid-lines represent results with only the $G^1_{\rm cw}$. The parameters are $G^1_{\rm cw}/2\pi = 0.64$ MHz, $G^2_{\rm cw}/2\pi = 0.71$ MHz, $J/2\pi = 1.5$ MHz, $\kappa_0/2\pi = 3$ MHz, $\kappa_\alpha/2\pi = 9$ MHz, $\kappa_\beta/2\pi = 4.2$ MHz, $\gamma_{\rm m,1}/2\pi = 22\times10^{-3}$ MHz, $\gamma_{\rm m,2}/2\pi = 62\times10^{-3}$ MHz, $\omega_{\rm m,1}/2\pi = 90.47$ MHz, and $\omega_{\rm m,2}/2\pi = 87.86$ MHz.**

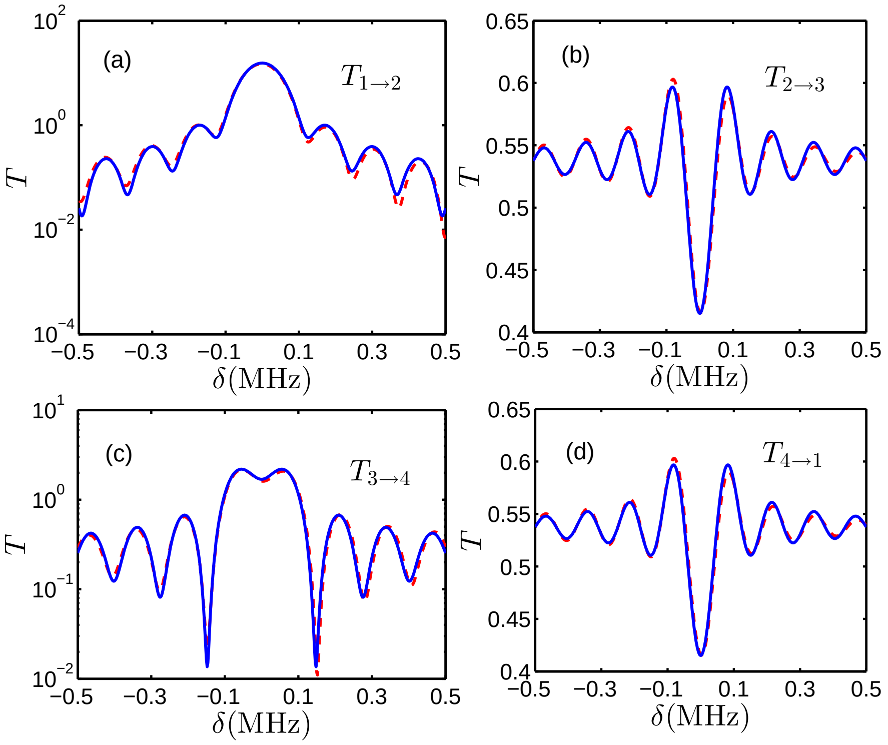

Supplementary Figure 7: **(a-d) Transmittance $T_{i\to i+1}$ as a function of the detuning $\delta = \omega - \omega_{\rm o}$. The red dashed-lines show the calculated transmission including the back-scattering and two mechanical modes. The blue solid-lines represent results only with the $G^1_{\rm cw}$. $G^1_{\rm cw}/2\pi = 0.55$ MHz and $G^2_{\rm cw}/2\pi = 0.61$ MHz, and the other parameters are the same as given in Supplementary Fig. 6.**

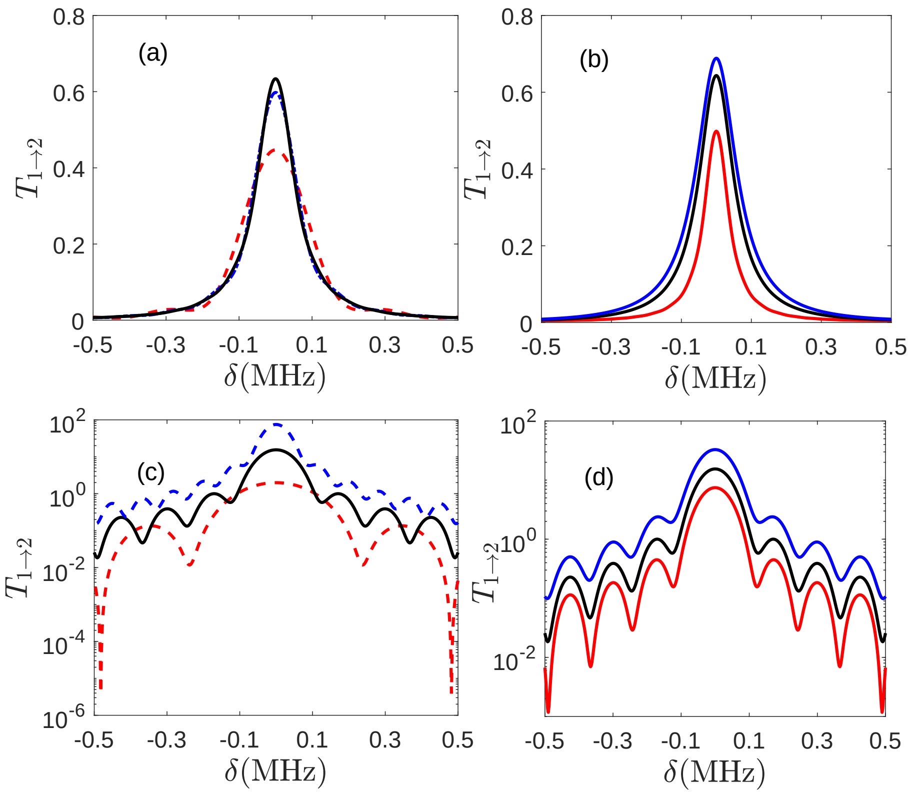

Supplementary Figure 8: **(Color online) Transmittance** $T_{1\to2}$ **as a function of the detuning** $\delta = \omega - \omega_o$**. These lines are the results of calculations using (a)** $C_{\rm cw} = 4.6$**,** $t_{\rm d} = 5$ **(red dashed-line),** 9 **(blue dashed-line),** 13 **(black solid-line) μs; (b)** $C_{\rm cw} = 2.8$ **(red line),** 4.6 **(black line),** 5.5 **(blue line) in the steady state; (c)** $C_{\rm cw} = -3.4$**,** $t_{\rm d} = 4$ **(red dashed-line),** 8 **(black solid-line),** 12 **(blue dashed-line) μs; (d)** $C_{\rm cw} = -2.8$ **(red line),** -3.4 **(black line),** -4 **(blue line) with** $t_{\rm d} = 8$ **μs. Other parameters are the same with the Supplementary Fig. 6.**

Supplementary Table 1: Transmission matrices. Transmission matrices are shown for the operation modes of the circulator, directional amplifier and add-drop filter, which correspond to $C_{\mathbf{cw}} = 4.6,\ -4$ and 0, respectively. The rows correspond to the respective input ports and the columns to the respective output ports.

	Transmission matrices
Circulator	$\begin{bmatrix} 0.0074 & 0.598 & 0.00221 & 0.0233 \\ 0.0109 & 0.00311 & 0.576 & 0.0012 \\ 0.00114 & 0.017 & 0.0006 & 0.743 \\ 0.581 & 0.0014 & 0.23 & 0.00066 \end{bmatrix}$
Directional Amplifier	$\begin{bmatrix} 1.48 & 32.8 & 0.66 & 20.0 \\ 0.0123 & 1.55 & 0.383 & 0.652 \\ 0.656 & 19.7 & 0.279 & 5.39 \\ 0.35 & 0.664 & 0.331 & 0.317 \end{bmatrix}$
Add-drop filter	$\begin{bmatrix} 0.0367 & 0.0076 & 0.0158 & 0.539 \\ 0.00977 & 0.041 & 0.548 & 0.0192 \\ 0.0203 & 0.53 & 0.0102 & 0.259 \\ 0.533 & 0.0182 & 0.25 & 0.00717 \end{bmatrix}$

Supplementary Table 2: Transmission matrices. Transmission matrices are shown for the operation modes of an ideal circulator, directional amplifier and add-drop filter. The rows correspond to the respective input ports and the columns to the respective output ports.

	Transmission matrices
Ideal Circulator	$\begin{bmatrix} 0 & 1 & 0 & 0 \\ 0 & 0 & 1 & 0 \\ 0 & 0 & 0 & 1 \\ 1 & 0 & 0 & 0 \end{bmatrix}$
Ideal Directional Amplifier	$\begin{bmatrix} 0.024 & 0.645 & 0.011 & 0.320 \\ 0.019 & 0.669 & 0 & 0.312 \\ 0.024 & 0.686 & 0.011 & 0.279 \\ 0 & 0.655 & 0.039 & 0.306 \end{bmatrix}$
Ideal Add-drop Filter	$\begin{bmatrix} 0 & 0 & 0 & 1 \\ 0 & 0 & 1 & 0 \\ 0 & 1 & 0 & 0 \\ 1 & 0 & 0 & 0 \end{bmatrix}$

Supplementary Note 1. Experimental setup

Device fabrication and characterization

A silica microsphere with a diameter of approximately 35 μm is fabricated using a CO_2 laser. Two tapered fibres are obtained by melting the standard SMF-28 silica fibre using a hydrogen flame. The silica microresonator and tapered fibres are kept in a clean chamber to avoid contamination. For the microsphere, there are two radial breathing modes around 90 MHz, as shown in the displacement power spectra (Supplementary Fig. **1**a). In the main text, we only focus on one mechanical mode with frequency $\omega_\mathrm{m}/2\pi = 90.47\,\mathrm{MHz}$ and dissipation rate $\gamma/2\pi = 22\,\mathrm{kHz}$. From our theoretical analysis, the other mechanical mode, with a frequency approximately 2.6 MHz away from our target mechanical mode does not affect the experimental results or theoretic analysis in this paper. Supplementary Figure **1**b shows the transmission spectrum $T_{1\to2}$ of the selected high-quality-factor whispering-gallery mode (WGM) without a control field. The coupling strength between CW and CCW whispering-gallery modes is fitted to be $J/2\pi = 1.5\,\mathrm{MHz}$, which is due to optical backscattering [1, 2].

Spectrum measurement

To measure the non-reciprocal transmission spectra of the optomechanical system, the signal should be launched in different propagation directions. Therefore, we should be able to switch the direction of the signal to different input ports. To accomplish this aim, we generate a signal light by modulating a weak probe beam at the modulation frequency (Ω), which is markedly red- or blue-detuned 250 MHz from the cavity resonance to avoid the disturbance of mechanical motion from the probe beam. One sideband, which is nearly resonant with the optical mode, serves as the signal light. Therefore, the path and propagation direction of the signal can be switched. The frequencies of the control, weak probe beam and signal lights used in the experiment are illustrated in Supplementary Figs. **2**a and b.

We use a network analyser to generate the modulation frequency of Ω and a heterodyne spectroscopic technique to measure the transmission spectra. If we directly focus on the beating between the weak probe beam and signal, we obtain the intracavity field instead of the transmission spectra [3–5]. To demonstrate the circulator and directional amplifier, we focus on the beating between the signal field and the local oscillator, which is the control

field or the separated control field. However, the beating frequency is near ω_{m}, which is different from the modulation frequency generated by the network analyser. Therefore, we upward shift the beating signal to Ω through frequency mixing with a signal generator operating at frequency ω_{SG}. This process can be realized by a single sideband modulator [6]. Supplementary Figure **2**c shows a schematic of the pulse sequence and the detection gate used in our experiment. The control and signal pulses are synchronized and have the same duration of $\tau = 10$ μs. In each cycle of the repeated experiments, the detection is followed by a procedure of laser locking to the cavity mode.

The detailed experimental setup is illustrated in Supplementary Fig. **3**. The strong control pulse and weak locking pulse are derived from the first-order diffraction of the laser passing through an acoustic-optic modulator (AOM1). The weak locking pulse is separated from these two pulses by AOM3 and fed into the photo-detector (D_5) [3]. The error signal can be obtained for the proportional-integral-derivative (PID) controller to lock the laser at the relevant frequency using the Pound-Drever-Hall technique when the weak locking pulse propagates through an electro-optic modulator (EOM1) with a modulation frequency $f = \omega_{\mathrm{m}}$. The weak probe beam is generated by a separate laser beam from the same laser and is derived from AOM2, the frequency of which is chosen according to the spectral positions shown in Supplementary Figs. **2**a and b. One of the sidebands generated from EOM2, which is driven by the output of a network analyser at frequency Ω, serves as the signal pulse. With the control pulse always incident from input port 1, the probe beam is controlled to launch into one of the four ports by flip mirrors and circulators and is then recombined with the control laser. The beating signals around ω_{m} on each detector (D_i, $i \in \{1, 2, 3, 4\}$) are mixed with a signal generator (SG2) to match the modulation frequency Ω. The transient behaviour of the optical signal transmission is probed using a network analyser operating in a time-gated detection mode with a resolution bandwidth of 1 MHz.

To calibrate the port-to-port transmission $T_{1\leftrightarrow 2}$ and $T_{3\leftrightarrow 4}$, we first measure the transmission spectra $I^0_{1\leftrightarrow 2}$ and $I^0_{3\leftrightarrow 4}$ with the silica microsphere moved far from the microfibres. These spectra are obtained with gated heterodyne detection under the same conditions as before. Then, the normalized transmission spectra can be calculated as the ratio of $I'_{1\leftrightarrow 2}/I^0_{1\leftrightarrow 2}$ and $I'_{3\leftrightarrow 4}/I^0_{3\leftrightarrow 4}$, where $I'_{1\leftrightarrow 2}$ and $I'_{3\leftrightarrow 4}$ are the raw transmission data. To calibrate the other spectra $T_{i\to j}$, the input spectra $I^0_{i\to j}$ of the weak probe beam are measured with the same heterodyne spectroscopic technique, prior to being sent into microfibre port i. Consider-

ing the insertion or absorption losses in the optical path, the transmission spectra $T'_{i\to j}$ are calibrated as $I'_{i\to j}/(\xi_{i,j}I^0_{i\to j})$, where $\xi_{i,j}$ is a constant that depends on the total power loss.

Supplementary Figure **5** shows both the experimental and corresponding calculated transmission spectra for all fibre ports. The control field is 5.8 mW for the red-detuned case and 2.9 mW for the blue-detuned case. It is worth noting that because the two radial breathing modes are close to each other, the transmission spectra show non-reciprocal spectral lineshapes at both separate frequencies, which coincide with the frequencies of the two mechanical modes. All of the theoretical transmission spectra shown in the main text and the Supplementary Materials are calculated using $C_{\text{cw}}/C'_{\text{cw}} = 2.28$, where C_{cw} is the cooperativity for the 90.47 MHz mechanical mode and C'_{cw} is for the 87.86 MHz mechanical mode. The detailed spectra around $\delta \sim 0$ for different experimental conditions are shown in Supplementary Fig. **5**. The transmission matrices plotted in Fig. 4 in the main text are explicitly listed in Supplementary Table 1.

Supplementary Note 2. Theory

Model

As shown in Supplementary Fig. **1**, the silica microsphere adapted in our experiment has two mechanical modes with frequency $\omega_{\text{m},j}$ $(j = 1,\ 2)$. In the main text, $\omega_{\text{m},1}$ is written as ω_{m}, $g_{0,1}$ is written as g_0, and $\gamma_{\text{m},1}$ is written as γ. The optical resonator supports two degenerate CW and CCW optical modes with the same frequency ω_{o}. Considering the control field sent into the CW optical mode, the full system can be described by the following Hamiltonian ($\hbar = 1$):

$$\begin{aligned} H =& \omega_{\text{o}} \left(c^\dagger_{\text{cw}}c_{\text{cw}} + c^\dagger_{\text{ccw}}c_{\text{ccw}}\right) + \sum_{j=1}^{2} \omega_{\text{m},j} m^\dagger_j m_j + J\left(c^\dagger_{\text{cw}}c_{\text{ccw}} + c_{\text{cw}}c^\dagger_{\text{ccw}}\right) \\ &+ \sum_{j=1}^{2} g_{0,j}(c^\dagger_{\text{cw}}c_{\text{cw}} + c^\dagger_{\text{ccw}}c_{\text{ccw}})\left(m_j + m^\dagger_j\right) + i\sqrt{\kappa_\alpha}\epsilon_{\text{c}}\left(c^\dagger_{\text{cw}}e^{-i\omega_{\text{c}}t} - c_{\text{cw}}e^{i\omega_{\text{c}}t}\right), \end{aligned} \tag{1}$$

where J is the photon-hopping interaction between the CW and CCW modes due to optical backscattering, $g_{0,j}$ is the single-photon optomechanical coupling rate for the j-th mechanical mode, ω_{c} is the control field frequency with amplitude ϵ_{c}, and $\kappa_{\alpha(\beta)}$ is the microfibre $\alpha(\beta)$ to microsphere coupling rate.

For a very weak coupling rate $g_{0,j} \ll \omega_{\mathrm{m},j},\ \kappa$, where $\kappa = \kappa_0 + \kappa_\alpha + \kappa_\beta$ is the total optical energy decay rate, the optomechanical interactions can be enhanced by the control laser ϵ_{c}. In the resolved-sideband limit, the nonlinear optomechanical coupling is linearized, which is given by:

$$
\begin{aligned}
H_{\mathrm{lin}} =& \Delta_{\mathrm{c}} \left(c_{\mathrm{cw}}^\dagger c_{\mathrm{cw}} + c_{\mathrm{ccw}}^\dagger c_{\mathrm{ccw}} \right) + \sum_{j=1}^{2} \omega_{\mathrm{m},j} m_j^\dagger m_j + J \left(c_{\mathrm{cw}}^\dagger c_{\mathrm{ccw}} + c_{\mathrm{cw}} c_{\mathrm{ccw}}^\dagger \right) \\
& + \sum_{j=1}^{2} \left(G_{\mathrm{cw}}^j c_{\mathrm{cw}}^\dagger + G_{\mathrm{ccw}}^j c_{\mathrm{ccw}}^\dagger + \mathrm{H.c.} \right) \left(m_j + m_j^\dagger \right), \qquad (2)
\end{aligned}
$$

Where $\Delta_{\mathrm{c}} = \omega_{\mathrm{o}} - \omega_{\mathrm{c}}$ and $G_{\mathrm{cw(ccw)}}^j = g_{0,j} \alpha_{\mathrm{cw(ccw)}}$. When the duration of the control pulse $\tau_{\mathrm{c}} \gg 1/\kappa$, the intracavity control field can be approximately treated as a constant field:

$$
\alpha_{\mathrm{cw}} = \frac{\sqrt{\kappa_\alpha} \epsilon_{\mathrm{c}}}{\kappa/2 + i\Delta_{\mathrm{c}} + \frac{J^2}{(\kappa/2 + i\Delta_{\mathrm{c}})}}, \qquad (3)
$$

$$
\alpha_{\mathrm{ccw}} = \frac{-iJ\alpha_{\mathrm{cw}}}{\kappa/2 + i\Delta_{\mathrm{c}}}. \qquad (4)
$$

When signal light is input into the system, the Hamiltonian becomes:

$$
\begin{aligned}
H_{\mathrm{sys}} =& \Delta_{\mathrm{c}} \left(c_{\mathrm{cw}}^\dagger c_{\mathrm{cw}} + c_{\mathrm{ccw}}^\dagger c_{\mathrm{ccw}} \right) + \sum_{j=1}^{2} \omega_{\mathrm{m},j} m_j^\dagger m_j + J \left(c_{\mathrm{cw}}^\dagger c_{\mathrm{ccw}} + c_{\mathrm{cw}} c_{\mathrm{ccw}}^\dagger \right) \\
& + \sum_{j=1}^{2} \left(G_{\mathrm{cw}}^j c_{\mathrm{cw}}^\dagger + G_{\mathrm{ccw}}^j c_{\mathrm{ccw}}^\dagger + \mathrm{H.c.} \right) \left(m_j + m_j^\dagger \right) \\
& + i \left(\sqrt{\kappa_\alpha} \varepsilon_{\mathrm{in}}^1 + \sqrt{\kappa_\beta} \varepsilon_{\mathrm{in}}^3 \right) \left(c_{\mathrm{cw}}^\dagger e^{-i(\omega - \omega_{\mathrm{c}})t} - c_{\mathrm{cw}} e^{i(\omega - \omega_{\mathrm{c}})t} \right) \\
& + i \left(\sqrt{\kappa_\alpha} \varepsilon_{\mathrm{in}}^2 + \sqrt{\kappa_\beta} \varepsilon_{\mathrm{in}}^4 \right) \left(c_{\mathrm{ccw}}^\dagger e^{-i(\omega - \omega_{\mathrm{c}})t} - c_{\mathrm{ccw}} e^{i(\omega - \omega_{\mathrm{c}})t} \right), \qquad (5)
\end{aligned}
$$

where $\varepsilon_{\mathrm{in}}^n$ is the weak signal light from the input port $n = 1,\ 2,\ 3,\ 4$.

Red sideband control

When the control field is red-detuned from the optical mode by a frequency of mechanical mode 1, that is, $\Delta_{\mathrm{c}} = \omega_{\mathrm{m},1}$, we can neglect the terms $c_{\mathrm{cw(ccw)}} m_j + \mathrm{H.c.}$ in the rotating wave approximation $\left(\left| G_{\mathrm{cw,ccw}}^j \right| \ll \omega_{\mathrm{m},j} \right)$. The dynamics of the optomechanical system can be

described by the following equations:

$$\frac{dc_{\text{cw}}}{dt} = -\left(\frac{\kappa}{2} + i\Delta_{\text{c}}\right) c_{\text{cw}} - i\left(G_{\text{cw}}^{1} m_1 + G_{\text{cw}}^{2} m_2\right) - iJc_{\text{ccw}} + \left(\sqrt{\kappa_{\alpha}}\varepsilon_{\text{in}}^{1} + \sqrt{\kappa_{\beta}}\varepsilon_{\text{in}}^{3}\right) e^{-i(\omega-\omega_{\text{c}})t}, \tag{6}$$

$$\frac{dc_{\text{ccw}}}{dt} = -\left(\frac{\kappa}{2} + i\Delta_{\text{c}}\right) c_{\text{ccw}} - i\left(G_{\text{ccw}}^{1} m_1 + G_{\text{ccw}}^{2} m_2\right) - iJc_{\text{cw}} + \left(\sqrt{\kappa_{\alpha}}\varepsilon_{\text{in}}^{2} + \sqrt{\kappa_{\beta}}\varepsilon_{\text{in}}^{4}\right) e^{-i(\omega-\omega_{\text{c}})t}, \tag{7}$$

$$\frac{dm_1}{dt} = -\left(\frac{\gamma_{\text{m},1}}{2} + i\omega_{\text{m},1}\right) m_1 - i\left(G_{\text{cw}}^{1*} c_{\text{cw}} + G_{\text{ccw}}^{1*} c_{\text{ccw}}\right), \tag{8}$$

$$\frac{dm_2}{dt} = -\left(\frac{\gamma_{\text{m},2}}{2} + i\omega_{m,2}\right) m_2 - i\left(G_{\text{cw}}^{2*} c_{\text{cw}} + G_{\text{ccw}}^{2*} c_{\text{ccw}}\right), \tag{9}$$

where $\gamma_{\text{m},j}$ is the damping rate of mechanical mode j.

Using the standard input-output theory $\varepsilon_{\text{out}} = \sqrt{\kappa_{\alpha,\beta}} c_{\text{cw}}\,(c_{\text{ccw}}) - \varepsilon_{\text{in}}$, we can obtain the output of every port. We define $T_{m\to n} = \left|\frac{\varepsilon_{\text{out}}^{n}}{\varepsilon_{\text{in}}^{m}}\right|^2$ as the transmittance of the ports from m to n. In Supplementary Fig. 6, we plot the conversion efficiency $T_{i\to i+1}$ as a function of the detuning δ. The red dashed-lines show the transmission spectra of the signal, where the system is under the control pulse with a gate delay of $t_{\text{d}} = 9$ µs, with $C_{\text{cw}}^{1} = 4.6$ and $C_{\text{cw}}^{2} = 2$, in which we define the cooperativity $C_{\text{cw}}^{j} \equiv 4\left|G_{\text{cw}}^{j}\right|^2/\kappa\gamma_{\text{m},j}$. We notice that $|G_{\text{ccw}}^{j}/G_{\text{cw}}^{j}| = \left|\frac{J}{\kappa/2+i\Delta_{\text{c}}}\right| \ll 1$; thus, the backscattering of the control field can be neglected in our experiments. If we only consider the detuning near $|\delta| \ll \omega_{\text{m},1} - \omega_{\text{m},2}$ when $\Delta_{\text{c}} = \omega_{\text{m},1}$, the optomechanical effect of mechanical mode 2 can also be ignored. The blue solid-lines in Supplementary Fig. **6** show the result with only the G_{cw}^{1} based on zero backscattering and single mechanical mode approximations. These results are almost the same as the red dashed-lines, indicating that the two approximations are valid for our experiments. For simplicity, we rewrite $C_{\text{cw}}^{1} = C_{\text{cw}}$, $\omega_{\text{m},1} = \omega_{\text{m}}$, and $\gamma_{\text{m},1} = \gamma$ in the following.

We note that the signal transmission spectra $T_{m\to n}$ depend on many parameters. For an intuitive understanding of the circulator, we solve the dynamical equations in the frequency domain, and the output of the system is obtained as $\varepsilon_{\text{out}} = R\varepsilon_{\text{in}}$, that is, $T_{m\to n} = |R_{nm}^{2}|$. The coefficient matrix is

$$R = \frac{1}{\lambda}\begin{pmatrix} -iJ\kappa_{\alpha} & \kappa_{\alpha}X_{\text{cw}} - \lambda & -iJ\sqrt{\kappa_{\alpha}\kappa_{\beta}} & \sqrt{\kappa_{\alpha}\kappa_{\beta}}X_{\text{cw}} \\ \kappa_{\alpha}X_{\text{ccw}} - \lambda & -iJ\kappa_{\alpha} & \sqrt{\kappa_{\alpha}\kappa_{\beta}}X_{\text{ccw}} & -iJ\sqrt{\kappa_{\alpha}\kappa_{\beta}} \\ -iJ\sqrt{\kappa_{\alpha}\kappa_{\beta}} & \sqrt{\kappa_{\alpha}\kappa_{\beta}}X_{\text{cw}} & -iJ\kappa_{\beta} & \kappa_{\beta}X_{\text{cw}} - \lambda \\ \sqrt{\kappa_{\alpha}\kappa_{\beta}}X_{\text{ccw}} & -iJ\sqrt{\kappa_{\alpha}\kappa_{\beta}} & \kappa_{\beta}X_{\text{ccw}} - \lambda & -iJ\kappa_{\beta} \end{pmatrix}, \tag{10}$$

where $\lambda = X_{\mathrm{cw}} X_{\mathrm{ccw}} + J^2$, in which

$$X_{\mathrm{cw}} = \frac{\kappa}{2} + i\left(\Delta_{\mathrm{c}} - \omega\right) + \frac{\left|G_{\mathrm{cw}}\right|^2}{\gamma/2 + i\left(\omega_{\mathrm{m}} - \omega\right)}, \tag{11}$$

$$X_{\mathrm{ccw}} = \frac{\kappa}{2} + i\left(\Delta_{\mathrm{c}} - \omega\right). \tag{12}$$

From the coefficient matrix, we find that $T_{m\to n} = \left|R_{nm}\right|^2$. Some matrix elements are the same, and we must determine the $T_{1\to2}$, $T_{2\to3} = T_{4\to1}$, and $T_{3\to4}$ for the circulator. When the frequency $\omega = \omega_{\mathrm{m}} = \Delta_{\mathrm{c}}$, we obtain $T_{1\to2} = \left|\frac{\kappa_{\alpha}}{\kappa(1+C_{\mathrm{cw}})/2+2J^2/\kappa} - 1\right|^2$, which approaches 1 with increasing cooperativity C_{cw}. $T_{2\to3} = \left|\frac{\sqrt{\kappa_{\alpha}\kappa_{\beta}}(1+C_{\mathrm{cw}})}{\kappa(1+C_{\mathrm{cw}})/2+2J^2/\kappa}\right|^2$ has a limit $\frac{2\sqrt{\kappa_{\alpha}\kappa_{\beta}}}{\kappa_{\alpha}+\kappa_{\beta}+\kappa_0}$ even for $C_{\mathrm{cw}} \gg 1$, implying that good circulator performance requires $\kappa_{\alpha} = \kappa_{\beta} \gg \kappa_0$. The signal transmission $T_{3\to4}$ has a form similar to that of $T_{1\to2}$.

Blue sideband control

By setting the detuning $\Delta_{\mathrm{c}} = -\omega_{\mathrm{m},1}$ (blue detuning), we can neglect the terms $c^{\dagger}_{\mathrm{cw(ccw)}} m_j$ + H.c., and the dynamics of the optomechanical system can be described by the following equations:

$$\frac{dc_{\mathrm{cw}}}{dt} = -\left(\frac{\kappa}{2} + i\Delta_{\mathrm{c}}\right) c_{\mathrm{cw}} - i\left(G^1_{\mathrm{cw}} m^{\dagger}_1 + G^2_{\mathrm{cw}} m^{\dagger}_2\right) - iJc_{\mathrm{ccw}} + \left(\sqrt{\kappa_{\alpha}}\varepsilon^1_{\mathrm{in}} + \sqrt{\kappa_{\beta}}\varepsilon^3_{\mathrm{in}}\right) e^{-i(\omega-\omega_{\mathrm{c}})t}, \tag{13}$$

$$\frac{dc_{\mathrm{ccw}}}{dt} = -\left(\frac{\kappa}{2} + i\Delta_{\mathrm{c}}\right) c_{\mathrm{ccw}} - i\left(G^1_{\mathrm{ccw}} m^{\dagger}_1 + G^2_{\mathrm{ccw}} m^{\dagger}_2\right) - iJc_{\mathrm{cw}} + \left(\sqrt{\kappa_{\alpha}}\varepsilon^2_{\mathrm{in}} + \sqrt{\kappa_{\beta}}\varepsilon^4_{\mathrm{in}}\right) e^{-i(\omega-\omega_{\mathrm{c}})t}, \tag{14}$$

$$\frac{dm^{\dagger}_1}{dt} = -\left(\frac{\gamma_{\mathrm{m},1}}{2} + i\omega_{\mathrm{m},1}\right) m_1 + i\left(G^{1*}_{\mathrm{cw}} c_{\mathrm{cw}} + G^{1*}_{\mathrm{ccw}} c_{\mathrm{ccw}}\right), \tag{15}$$

$$\frac{dm^{\dagger}_2}{dt} = -\left(\frac{\gamma_{\mathrm{m},2}}{2} + i\omega_{\mathrm{m},2}\right) m_2 + i\left(G^{2*}_{\mathrm{cw}} c_{\mathrm{cw}} + G^{2*}_{\mathrm{ccw}} c_{\mathrm{ccw}}\right). \tag{16}$$

In Supplementary Fig. **7**, we plot the signal transmission spectra $T_{i\to i+1}$ as a function of the detuning δ during the control pulse with a gate delay of $t_{\mathrm{d}} = 8$ μs. The blue solid-lines show the results with only G^1_{cw}, which are almost the same as the red dashed-lines considering both the backscattering of control field and the second mechanical mode, where the cooperativity $C^1_{\mathrm{cw}} = -4\left|G^1_{\mathrm{cw}}\right|^2/\kappa\gamma_{\mathrm{m},1} = -3.4$. It is obvious that our approximation is also valid for blue detuning.

Bandwidth and sinc-function like oscillations

In our experiments, we observe the transmission spectra from the microcavity, which are shown in Figs. 2 and 3. In order to avoid the thermal effect, the control laser and signal we used are pulses instead of continuous waves, and the signal transmission spetra are shown as

$$\begin{aligned} c_{\mathrm{cw}}(\delta) &\approx \frac{-\sqrt{\kappa_{\mathrm{in}}}\varepsilon_{\mathrm{in}}}{i\delta-\frac{\kappa}{2}(1+\frac{C_{\mathrm{cw}}}{1-i\delta/\frac{\gamma}{2}})}\left[1+\frac{2C_{\mathrm{cw}}\exp(i\delta t)A(t)}{1-i\delta/\frac{\gamma}{2}}\right] \\ &= \frac{-\sqrt{\kappa_{\mathrm{in}}}\varepsilon_{\mathrm{in}}\left[\left(\frac{\gamma}{2}+\gamma A(t)C_{\mathrm{cw}}\cos(\delta t)\right)-i\left(\delta+\gamma A(t)C_{\mathrm{cw}}\sin(\delta t)\right)\right]}{\left(\frac{\gamma}{2}-i\delta\right)i\delta-\frac{\kappa}{2}\left[\frac{\gamma}{2}(1+C_{\mathrm{cw}})-i\delta\right]}, \end{aligned} \tag{17}$$

where we have ignored the back-scattering J between the traveling optical modes and $A(t) = \exp(-(\kappa+\gamma)/4)\sinh\left[\frac{t}{2}\sqrt{\left(\frac{\kappa+\gamma}{2}\right)^2-\kappa\gamma(1+C_{\mathrm{cw}})}\right]$. In order to explain the sinc-function like oscillations, we show the theoretical results for the transmission spectra at the longer control pulses with different gate delay in Supplementary Figs. 8a and c. It is obvious that the additional side-peaks become smaller amplitude and shorter period, and the single central resonance has the narrower effective linewidth and taller peak with the longer gate delay. For $C_{\mathrm{cw}} > -1$, only when the interaction time $t \gg (\kappa+\gamma)/\left[\kappa\gamma(1+C_{\mathrm{cw}})\right]$, we have the transient modification $A(t) \to 0$, which leads to disappearing of these additional peaks, and especially when blue-detuned control field, it takes longer time to establish the steady state for optomechanically induced amplification. In Supplementary Fig. 8b, the steady-state spectrum is obtained as $c_{\mathrm{cw}}(\delta) = \frac{-\sqrt{\kappa_{\mathrm{in}}}\varepsilon_{\mathrm{in}}}{i\delta-\frac{\kappa}{2}(1+\frac{C_{\mathrm{cw}}}{1-i\delta/\frac{\gamma}{2}})}$, and the bandwidth is $\gamma(1+C_{\mathrm{cw}})$, which can be improved by increasing the the control field power for $C_{\mathrm{cw}} > 0$. However, for the blue-detuned control $C_{\mathrm{cw}} < -1$, the system can not reach steady, which is shown in Supplementary Figs. 8c and d. The bandwidth can be tuned by both the duration t_{d} and strength of the control power, which is also explained by the Supplementary Equation 17. In conclusion, the bandwith can be improved by increasing the strength of the control power with the shorter duration t_{d}.

Ideality

In this study, we quantify the performance of the circulator using the ideality I, which describes the overlap between the normalized transmission matrix $T^{\mathrm{N}}_{i\to j} = T_{i\to j}/\eta_i$ and that of the corresponding ideal device $T^{\mathrm{I}}_{i\to j}$, which is explicitly listed in Supplementary Table 2.

Here, $I = \frac{\mathrm{Tr}[T^{\mathrm{N}}_{i\to j} \cdot T^{\mathrm{I}}_{i\to j}]}{\mathrm{Tr}[T^{\mathrm{I}}_{i\to j} \cdot T^{\mathrm{I}}_{i\to j}]} = 1 - \frac{1}{8}\sum_{i,j}\left|T^{\mathrm{N}}_{i\to j} - T^{\mathrm{I}}_{i\to j}\right|$, reflects the overall performance considering every port-to-port transmission, where $I = 1$ for an ideal device. $\eta_i = \sum_j T_{i\to j}$ is the total transmission for the signal field entering port i ($1 - \eta_i$ is due to the intrinsic loss of the device). Note that the transmission matrix of the ideal directional amplifier is the steady-state solution with $C^1_{\mathrm{cw}} = -1.0$. The isolation $I_i = 10\log(T_{i\to j}/T_{j\to i})$ and insertion loss $L_i = -10\log(T_{i\to j})$ can also describe the performance of the circulator for each port i, yielding $(I_i) = (17.4, 15.3, 5.1, 13.4)$dB and $(L_i) = (2.2, 2.4, 1.3, 2.4)$dB for a control power of 7.8 mW, corresponding to $C_{\mathrm{cw}} = 4.6$.

Supplementary References

---

[1] Gorodetsky, M. L., Pryamikov, A. D. & Ilchenko, V. S., Rayleigh scattering in high-Q microspheres, J. Opt. Soc. Am. B **17**, 1051 (2000).

[2] Kippenberg, T. J., Spillane, S. M. & Vahala, K. J. Modal coupling in traveling-wave resonators, Opt. Lett. **27**, 1669 (2002).

[3] Shen, Z., Dong, C.-H., Chen, Y., Xiao, Y.-F., Sun, F.-W. & Guo, G.-C. Compensation of the Kerr effect for transient optomechanically induced transparency in a silic a microsphere, Opt. Lett. **41**, 1249 (2016).

[4] Weis, S., Riviere, R., Deleglise, S., Gavartin, E., Arcizet, O., Schliesser, A. & Kippenberg, T. J., Optomechanically induced transparency, Science **330**, 1520–3 (2010).

[5] Safavi-Naeini, H., Mayer Alegre, T. P., Chan, J., Eichenfield, M., Winger, M. , Q. Lin, Hill, J. T., Chang, D. E. & Painter, O., Electromagnetically induced transparency and slow light with optomechanics, Nature **472**, 69–73 (2011).

[6] Kim, J., Kuzyk, M. C., Han, K., Wang, H. & Bahl, G. , Non-reciprocal Brillouin scattering induced transparency, Nat. Phys. **11**, 275–280 (2015).
